# Introducing palmfungi.org, an integrated fungal-host data platform

**DOI:** 10.3897/BDJ.12.e126553

**Published:** 2024-10-02

**Authors:** Yinru Xiong, Manawasinghe S. Ishara, Kevin D. Hyde, Joanne E. Taylor, Alan Phillips, Diana Santos Pereira, Li Lu, Sheng-Nan Zhang, Ausana Mapook, Biao Xu

**Affiliations:** 1 Center of Excellence in Fungal Research, Chiang Rai, Thailand Center of Excellence in Fungal Research Chiang Rai Thailand; 2 School of Science, Mae Fah Luang University, Chiang Rai, Thailand School of Science, Mae Fah Luang University Chiang Rai Thailand; 3 Innovative Institute for Plant Health, Key Laboratory of Green Prevention and Control on Fruits and Vegetables in South China, Ministry of Agriculture and Rural Affairs, Zhongkai University of Agriculture and Engineering, Guangzhou, China Innovative Institute for Plant Health, Key Laboratory of Green Prevention and Control on Fruits and Vegetables in South China, Ministry of Agriculture and Rural Affairs, Zhongkai University of Agriculture and Engineering Guangzhou China; 4 CAS Key Laboratory for Plant Diversity and Biogeography of East Asia, Kunming Institute of Botany, Chinese Academy of Science, Kunming, China CAS Key Laboratory for Plant Diversity and Biogeography of East Asia, Kunming Institute of Botany, Chinese Academy of Science Kunming China; 5 Department of Botany and Microbiology, College of Science, Riyadh, Saudi Arabia Department of Botany and Microbiology, College of Science Riyadh Saudi Arabia; 6 Royal Botanic Garden Edinburgh, Edinburgh EH3 5LR, United Kingdom Royal Botanic Garden Edinburgh Edinburgh EH3 5LR United Kingdom; 7 Biosystems and Integrative Sciences Institute, Faculdade de Ciências da Universidade de Lisboa, Lisbon, Portugal Biosystems and Integrative Sciences Institute, Faculdade de Ciências da Universidade de Lisboa Lisbon Portugal; 8 Center for Yunnan Plateau Biological Resources Protection and Utilization, College of Biological Resource and Food Engineering, Qujing Normal University, Qujing, China Center for Yunnan Plateau Biological Resources Protection and Utilization, College of Biological Resource and Food Engineering, Qujing Normal University Qujing China; 9 School of Life Science and Technology, Center for Informational Biology, University of Electronic Science and Technology of China, Chengdu, China School of Life Science and Technology, Center for Informational Biology, University of Electronic Science and Technology of China Chengdu China

**Keywords:** Arecaceae, Ascomycota, Basidiomycetes, website, fungal host interaction

## Abstract

Palm fungi are a diverse and unique group mostly found on Arecaceae hosts. They have been studied for approximately 200 years resulting in a large number of known fungal species representing over 700 genera. The timeline of palm fungal studies could be roughly divided into three phases, based on the methods and frequency of reports. They are the “Historical palm fungi era”, “Classical palm fungi era” and “Molecular palm fungi era”. In the first two periods, the identification of palm fungi was based on morphology, which resulted in a considerable number of morphological species scattered across the data in books, monographs and papers. With the advancement of molecular techniques, studies on palm fungi accelerated. A large number of new species were introduced in the molecular era, especially from Asia, including China and Thailand. However, there is a necessity to link these three generations of studies into a single platform combining data related to host factors, geography and utilisation. Herein, we introduce the palm fungi website: https://palmfungi.org, an integrated data platform for interactive retrieval, based on palm and fungal species. This website is not only a portal for the latest, comprehensive species information on palm fungi, but also provides a new platform for fungal researchers to explore the host-specificity of palm fungi. Additionally, this study uses palmfungi.org and related data to briefly discuss the current status of research on the distribution of palm fungi populations, showing how palmfungi.org links fungi with their palm hosts. Furthermore, the website will act as a platform for collaboration amongst taxonomists, plant pathologists, botanists, ecologists and those who are interested in palms and their relationship with ecological sustainability.

## Introduction

To document the diversity and distribution of different organismal taxa, it is important to set out recording and standardised monitoring programmes, often with the help of citizen scientists (Mueller et al. 2004, Halme et al. 2012, Haelewaters et al. 2024). fungi are an ancient and thriving group of organisms that play a vital role in natural ecosystems (Stubblefield et al. 1985, Taylor et al. 2014, Xie et al. 2023). As early as the beginning of the 20^th^ century, evidence of fungi living in the Tertiary period was found in fossils and, since then, a large number have been documented (Kidston and Lang 1921, Edwards 1922). In the 21^st^ century, the description of new species and recording of fungi have been occurring at a higher frequency (Hawksworth and Lücking 2017, Fisher et al. 2020). Over the centuries, mycologists have been working to study the diversity of fungi. The "*Sylloge Fungorum*" published by Pier Andrea Saccardo in 1882 stimulated extensive research and discussion on fungal diversity amongst modern mycologists (Bolman 2023). This discussion continues to this day, with Hawksworth (2001) suggesting that the number of fungi might be 1.5 million species, O’Brien et al. (2005) estimating there might be 3.5 to 5.1 million species, while Schmit and Mueller (2007) suggest that there may be as few as 712,000 species. According to a more recent study by Niskanen et al. (2023), the current estimates for global fungal richness ranges between 2 and 3 million species, with a “best guess” at 2.5 million of which only about 150,000 fungi have been described (Species Fungorum 2024; http://www.speciesfungorum.org). There are three major ecological strategies of fungi: saprotrophic, parasitic and mutualistic (Willis 2018). To adapt to different ecological setting, fungi have a variety of life modes, including endophytes, pathogens or saprobes and, depending on environmental conditions, they may be able to shift from one lifestyle to another, such as endophytes becoming pathogens (Hyde et al. 2014, Bhunjun et al. 2023). These diverse lifestyles allow fungi to have a wide range of hosts, from animals to plants. Amongst these, the relationship between fungi and plants has been well studied (Senanayake et al. 2020, Jayawardena et al. 2021, Maharachchikumbura et al. 2021, Manawasinghe et al. 2021).

Arecaceae, which is commonly known as palms, ranks fifth in species richness amongst monocots families (Chen et al. 2022). According to APG IV (Angiosperm Phylogeny Group et al. 2016), Arecaceae contains five subfamilies, namely Arecoideae, Calamoideae, Ceroxyloideae, Coryphoideae and Nypoideae. This family is one of the most morphologically diverse angiosperm groups, with 181 genera and approximately 2600 species (Dransfield et al. 2005, Dransfield et al. 2008, Baker and Dransfield 2016). To better record palm species data, Kissling et al. (2019) established the PalmTraits 1.0 database and a species information retrieval website palmweb (https://palmweb.org). An increasing number of palm fungi are also being continuously recorded and reported. As represented by monographs of Fröhlich and Hyde (2000), Hyde et al. (2000) and Taylor and Hyde (2003), a large number of palm fungi have been recorded, laying a solid foundation for subsequent research on fungal diversity on palm plants and exploring the diversity of the entire fungal population.

According to the database of Farr et al. (2021), there are about 9500 records of palm fungi, distributed within 99 palm genera. The diversity of palm fungi is extremely broad, covering almost all major fungal taxa (Taylor et al. 1999, Fröhlich and Hyde 2000). fungi on palms may be saprobes (Taylor et al. 1999, Fröhlich and Hyde 2000), pathogens (Douanla-Meli and Scharnhorst 2021, Xiong et al. 2022b), endophytes (Taylor et al. 1999) or epiphytes (Marasinghe et al. 2022, Marasinghe et al. 2023). Earlier focal studies by Pinruan et al. (2007) reported fungi on the *Licualalongicalycata*, while Pinnoi et al. (2006) reported those on the *Eleiodoxaconferta*, both from a peat swamp in southern Thailand. Recent introductions of new saprobic taxa on palms are by Konta et al. (Konta et al. 2020, Konta et al. 2021, Konta et al. 2023b) and Xiong et al. (2022a), who have expanded our understanding of palm-associated fungal diversity. Zheng et al. (2017), Pandian et al. (2021) and Xiong et al. (2022b) reported pathogenic fungi on palms and Guo et al. (1998) and Mahmoud et al. (Mahmoud et al. 2016, Mahmoud et al. 2017) reported endophytic fungi on palms. However, the research on palm fungi is scattered, lacking a comprehensive and up to date source of knowledge to precisely document them. Many asexual palm fungi have also not been linked to their sexual morphs (Hyde et al. 2011, Wijayawardene et al. 2017). To overcome these gaps, we proposed a website dedicated to palm fungi which will be an interactive platform for mycologists, as well the those who are interested in palms in general.

In recent years, web pages dedicated to fungal groups have become important resources to retrieve information. Other than the traditional fungal databases dedicated to fungal classification, several new databases were introduced as websites. Taxa depository databases include MycoBank (Crous et al. 2004) and Index Fungorum (Index Fungorum 2024, https://www.indexfungorum.org). Other generally important databases are Facesoffungi (Jayasiri et al. 2015, https://www.facesoffungi.org), Outlineoffungi (Wijayawardene et al. 2020, Wijayawardene et al. 2022b, https://www.outlineoffungi.org) and Fungalpedia (Hyde et al. 2023). In addition, there are several recently introduced fungal web pages which are dedicated to specific hosts, ecosystems and localities, allowing researchers to easily access and cite relevant data. A few examples are dothideomycetes.org (Pem et al. 2019) dedicated to Dothideomycetes, botryosphaeriales.org (Wu et al. 2021) dedicated to Botryosphaeriales, coelomycetes.org (Huanraluek et al. 2021) dedicated to Coelomycetes, Italian microfungi.org (Wijesinghe et al. 2021) dedicated to fungi associated with Italian flora, Soilfun.org on soil-inhabiting Ascomycota (Yasanthika et al. 2023) and Beeltehangers.org (de Groot et al. 2024) dedicated to explore spatiotemporal trends of *Hesperomycesharmoniae*.

As a timely and significant addition to the palm fungal studies, herein we introduce palmfungi.org an online platform dedicated to fungi associated with various palm species worldwide. This study uses palmfungi.org and related data to briefly discuss the current status of research on the distribution of palm fungi populations, showing how palmfungi.org serves as a bridge between the available data on fungi and their palm hosts, providing researchers with a sustainable platform for fungal information. In addition, this website will be the global consortium for studies on various aspects of palm fungi.

## Why do we need palmfungi.org?

Palm fungi research and records have a long history. Based on the language and frequency of reports, Pereira and Phillips (2023) divide the history of palm fungus research into three phases: 1880s – 1920s, 1920s – 1990s and 1990s – the present. However, we re-define the time ranges of these three phases after adding the standard based on the method of fungal identification. We named the first phase as “Historical palm fungi era”, which has been reported sporadically using short Latin paragraphs and relatively subjective identification methods, from the 1820s to 1990s. The second phase we named as “Classical palm fungi era”, which was led by K.D. Hyde and co-authors using standard morphological methods to identify fungi on palms, from the 1990s to around 2005. The third phase is named “Molecular palm fungi era”, which combined molecular analysis with morphology for standardised fungal identification, started around 2005 to continues today. Taylor and Hyde (2003) reported the classification of a large amount of palm fungi, prompting researchers to pay more attention to how to collect and integrate palm fungi. However, the decentralised reporting palm fungi means that data cannot be collected and integrated simultaneously, which mightcause misjudgement of the diversity of palm fungi. Most of the fungal species introduced in the early 20^th^ century lacked molecular data or living cultures. Furthermore, these data are mostly published in printed books and not frequently referred to or cited. Palmfungi.org as an integrated sustainable data platform will facilitate solving this issue. This website will provide researchers with concise, relatively complete information on palm fungal species and as a directory of palm fungal diversity and host-specificity (Zhou and Hyde 2001).

In addition to connecting the history of palm fungi with modern data, this website also correlates palm fungi and palm plant taxa by citing the identification and classification of palms by Kissling et al. (2019) and Yao et al. (2023). In recent years, the research on palms has not been limited to the classification and identification of species. Most people have focused on the application and uses of palms (Khan et al. 2023). By establishing itself as a data bridge between available data on fungi and palms, Palmweb (https://palmweb.org) has the potential to become an important tool in applied research.

## What is palmfungi.org?

**Figure 1.**
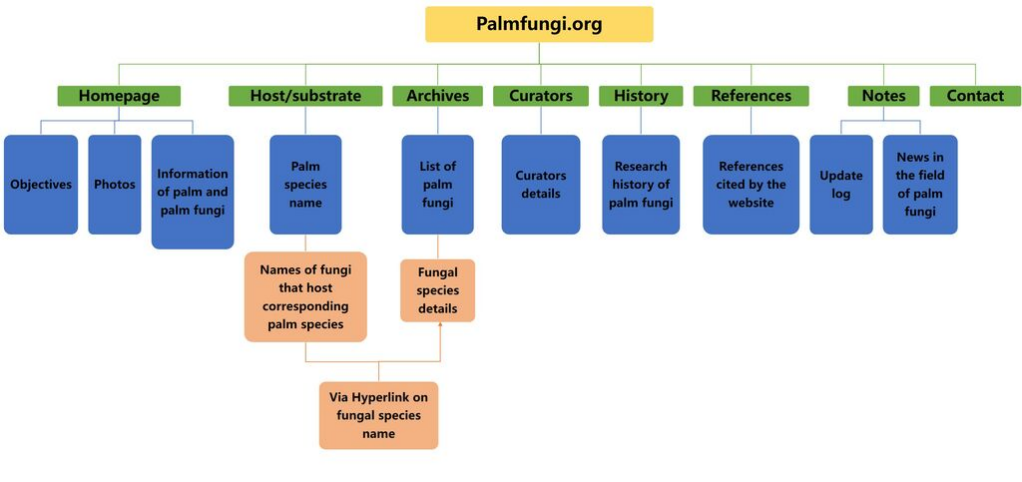
Palmfungi.org operation process.

The role of palmfungi.org is to establish a retrieval database, based on fungi species that use palms as hosts. The operation process of the website is shown in Fig. 1. In terms of host data, we listed the names of all known species of Arecaceae, according to Kissling et al. (2019) and Palmweb (2023) and the taxonomic status of Arecaceae species will be kept updated. In terms of fungal classification, we followed the “Outline of fungi” (Wijayawardene et al. 2022b, https://www.outlineoffungi.org). Meanwhile, we have added the vast majority of fungal species names from palms and dedicated entries for some of the species for which information is available. In addition, we implemented interactive searches for palm plant species and fungal species through hyperlinks and dual-function tags.

We will further refine the summary including the order, family, genus of fungi and other important data. Readers can click on relevant links from the palm fungi website which will redirect to the other databases including “Faces of fungi” (Jayasiri et al. 2015, http://www.facesoffungi.org); “Onestopshop fungi” (Jayawardena et al. 2019, https://onestopshopfungi.org); “Marine fungi” (Jones et al. 2019, http://marinefungi.org); “Freshwater fungi” (Calabon et al. 2020, http://freshwaterfungi.org); “Sordariomycetes” (Bundhun et al. 2020, https://sordariomycetes.org); “Fungal Genera” (Monkai et al. 2020, https://www.fungalgenera.org); “Outline of fungi” (Wijayawardene et al. 2020, https://www.outlineoffungi.org) and "gmsmicrofungi" (Chaiwan et al. 2021, http://gmsmicrofungi.org).

## Association with plant classification

According to APG IV (Angiosperm Phylogeny Group et al. 2016), Arecaceae (Palm) is the largest family within the order Arecales. Kissling et al. (2019) reported that there are 181 genera and nearly 2,600 species of palms. Despite this, taxonomic studies on palm plants are still common in recent years (Bellot et al. 2020, Helmstetter et al. 2020, Hodel et al. 2021, Jiménez et al. 2021, Eiserhardt et al. 2022). For fungi, especially saprophytic fungi, confirming the host species is a key step in taxonomic identification (Senanayake et al. 2020). Palmfungi.org cites identification and classification information of palm trees by Kissling et al. (2019) and Yao et al. (2023) and keeps it updated. Thus, Palmfungi.org will be a useful platform to solve this challenge and, together with Palmweb (https://palmweb.org), it will build a data bridge between the fungi and plants.

## Construction

Following the Outline of Ascomycetes (Wijayawardene et al. 2022b), all fungi using palm plants as hosts are included in the database. The database of palmfungi.org will be updated periodically as new information becomes available. Outlines, detailed descriptions and relevant information on each entry on the website will be carefully verified by the expert curators (Table 1).

## Database interface and visualisation

Palmfungi.org is an online fungi-palm interactive retrieval platform that compiles published information based on the taxonomy of fungi that host palm plants. The functions of the website are diverse, the interface is simple and user-friendly. The website consists of eight tabs; home, hosts substrate, archives, curators, history, references, notes and contact details as each tab with different functions. In addition, there is a right toolbar for searching and displaying recent updates. Finally, the lower border for displaying copyright and entry content to display fungal species details.

In total, there are 11 different features and functional details of the website. This includes the “a species entry” which is the building block of this website. For all these sections, detailed descriptions are as follows:

### A species entry

**Figure 2.**
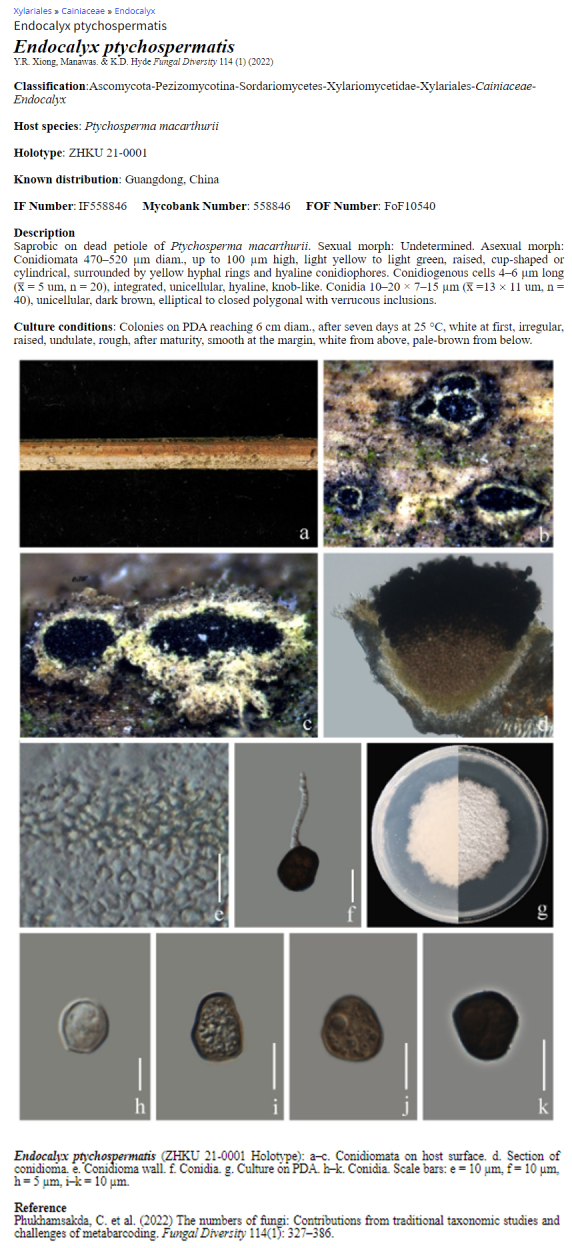
A single species entry of the palm web.

A single entry comprises species name, taxonomic classification database numbers such as Faces of fungi number, Index Fungorum and MycoBank number, description, host, distribution, coloured photo plate and illustrations, culture characteristics and references (Fig. 2).

### Right toolbar

**Figure 3.**
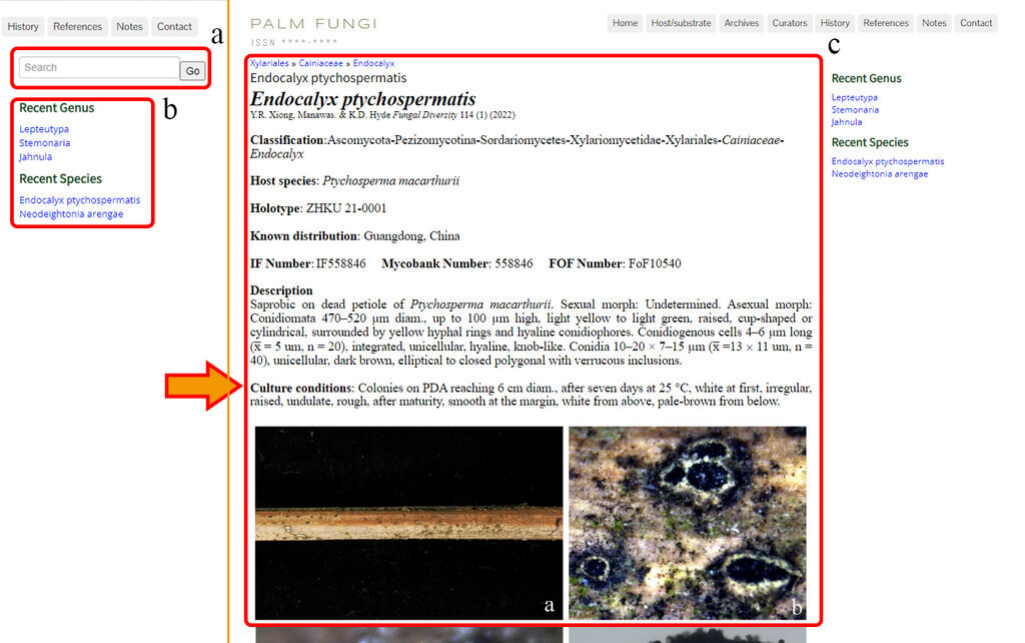
Right toolbar and search result: **a** Search toolbar; **b** Recently updated news, recent genera and species; **c** The entry details the interface of fungi species.

The right toolbar consists of three sections, the search toolbar (a), recent genus and recent species (b), are fixed on the right side of the entire website (Fig. 3). The fungal genus or taxon of interest can be entered in the search toolbar. Then a pop-up window will prompt the target fungus, including its taxonomic level. Clicking on the corresponding entry name will lead you to the species entry interface.

### Lower border

**Figure 4.**
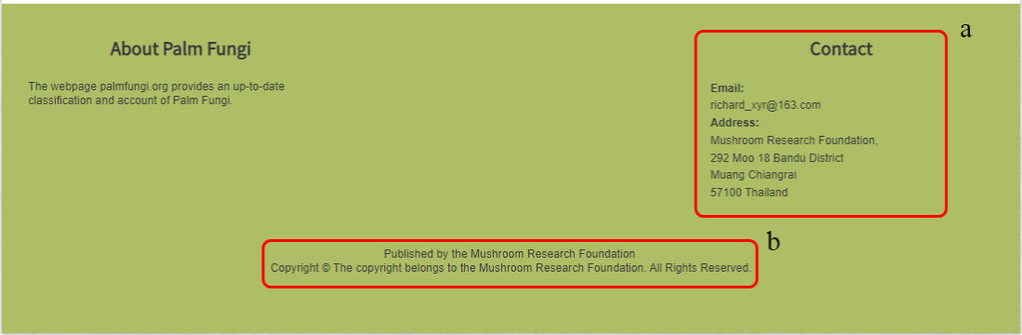
Bottom border: **a** Contact details; **b** Publisher and copyright information.

This information shows contact details and copyright ownership (Fig. 4).

### Homepage

**Figure 5.**
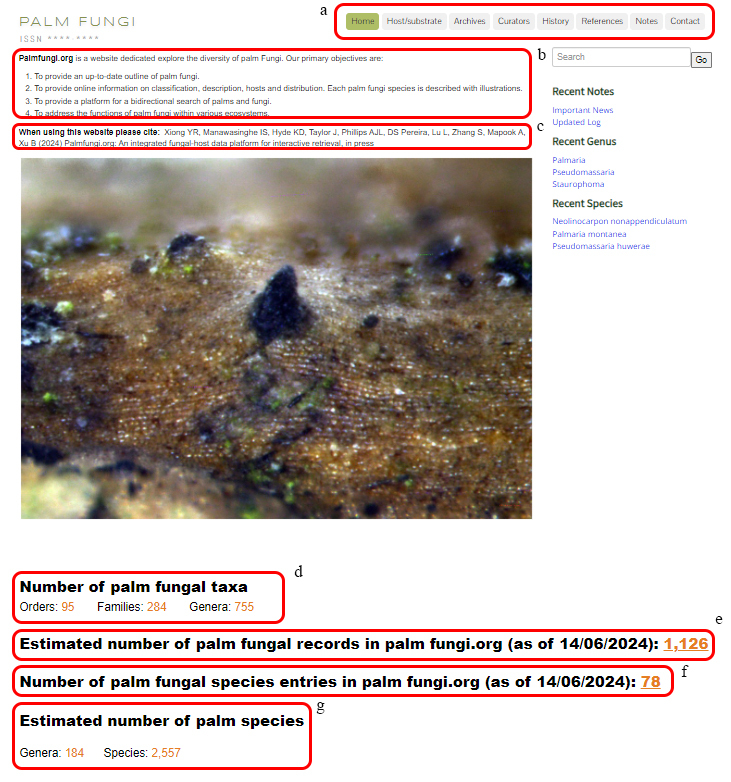
Homepage: **a** Headers; **b** Objectives of the website; **c** Citation of the website; **d** Number of palm fungi (including Order, family and genus); **e** Number of palm fungi records (Documented on the website after screening); **f** Number of palm fungi species entries; **g** Number of palm species (including each classification level).

The homepage (Fig. 5) shows the objectives of the website and the general information of the web (the function menu includes the search toolbar, home page, host/substrate, archive, curator, history, references, notes, contact). In addition, in Fig. 5c, it shows users the reference materials when citing this website. In addition, the number of palm fungi (including each classification level) and the number of palm species (including each classification level) currently included on the website are also shown.

### Host/Substrate

**Figure 6.**
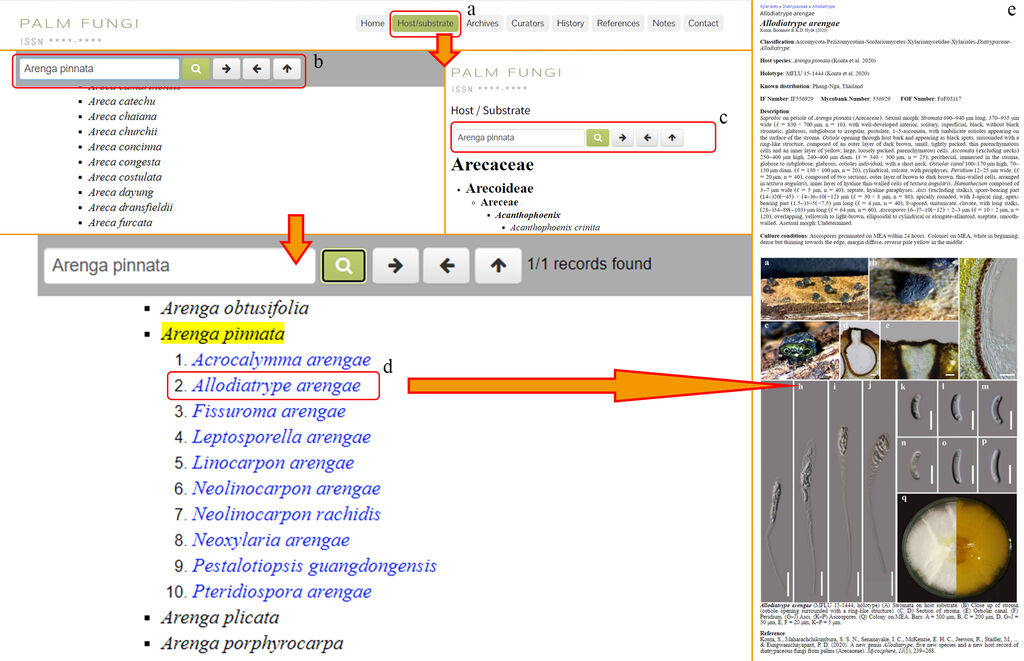
Host/Substrate: **a** Host/Substrate tag; **b** Frozen search bar; **c** Search bar; **d** Palm fungus species names with hyperlinks; **e** The entry details the fungal species that host this palm species.

By entering this tab, users can obtain information regarding all currently-known palm species based on their classification (https://palmweb.org) (Fig. 6). Users can intuitively find the fungal species and quantities reported on each palm species. At the same time, each palm species provides entry to the fungal species that host this palm species. In addition, there is also a search bar frozen at the top of the page, where you can enter the palm genus or taxon of interest and the fungus genus or taxon and the page will automatically lock to the target entry. Clicking on the corresponding entry name will guide users to the entry's detailed information interface.

### Archives

**Figure 7.**
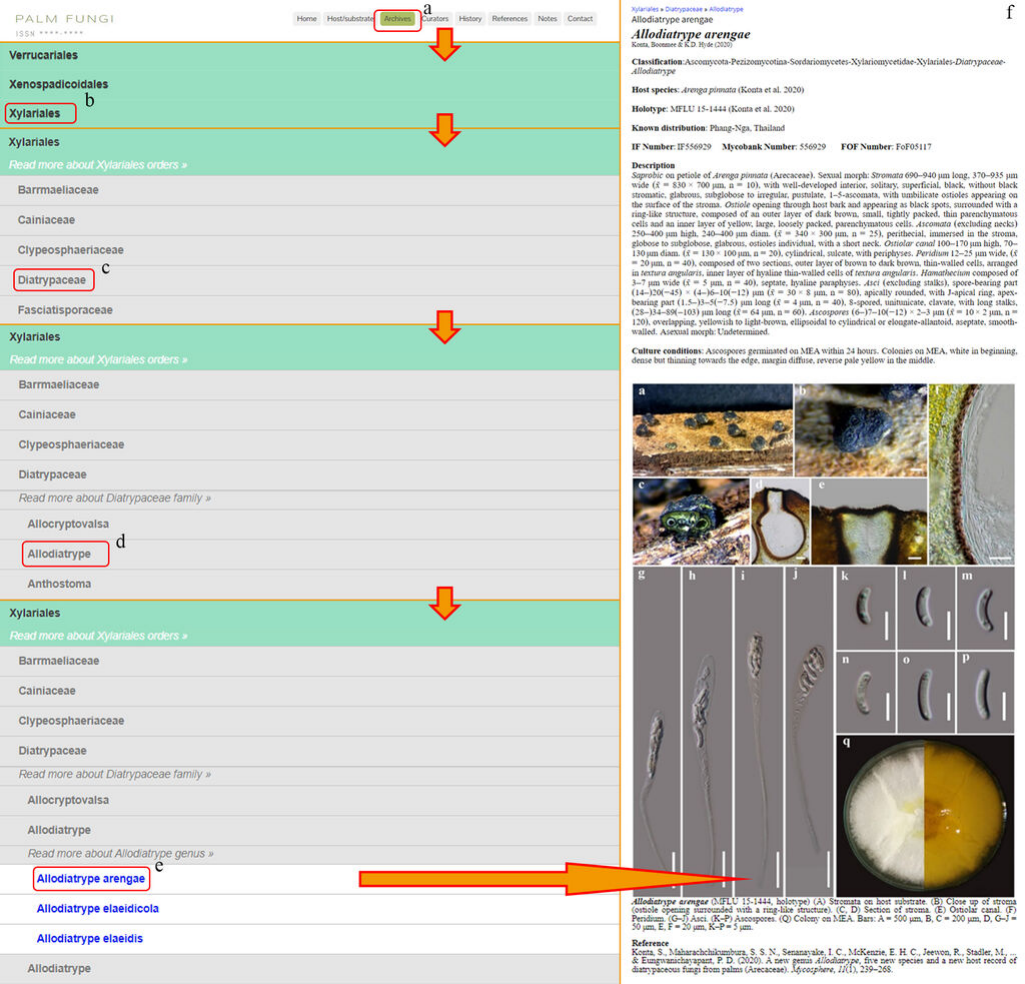
Archive: **a** Archive tag; **b** Order list; **c** Family list; **d** Genus list; **e** Species list; **f** The entry details the fungal species.

Provides users with a relevant list of palm fungi at various classification levels (highest classification level is order, lowest classification level is species) (Fig. 7). By clicking on the relevant term, the user is presented with options for "Read more about this entry" and a list of sub-categories.

### Curators

**Figure 8.**
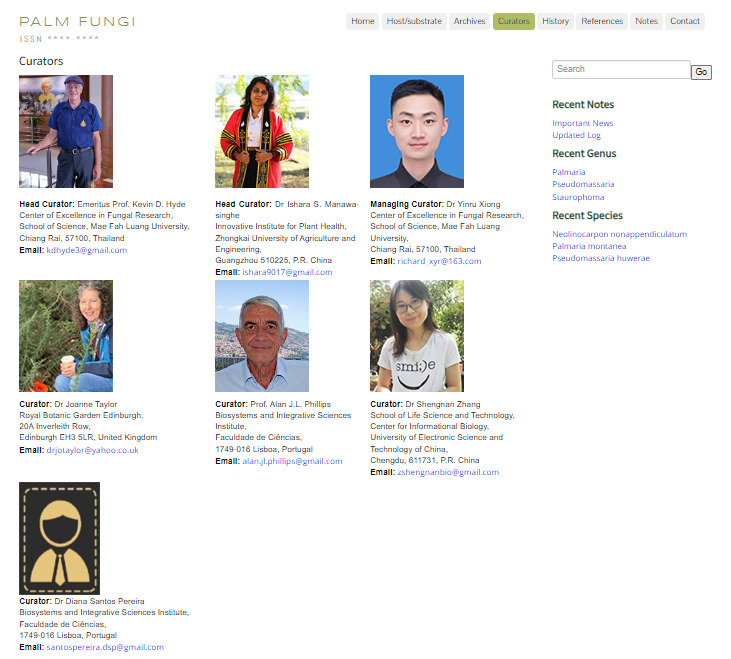
Curators: Website curator information.

Provides the contact information and affiliation of the website curators (Fig. 8).

### History

**Figure 9.**
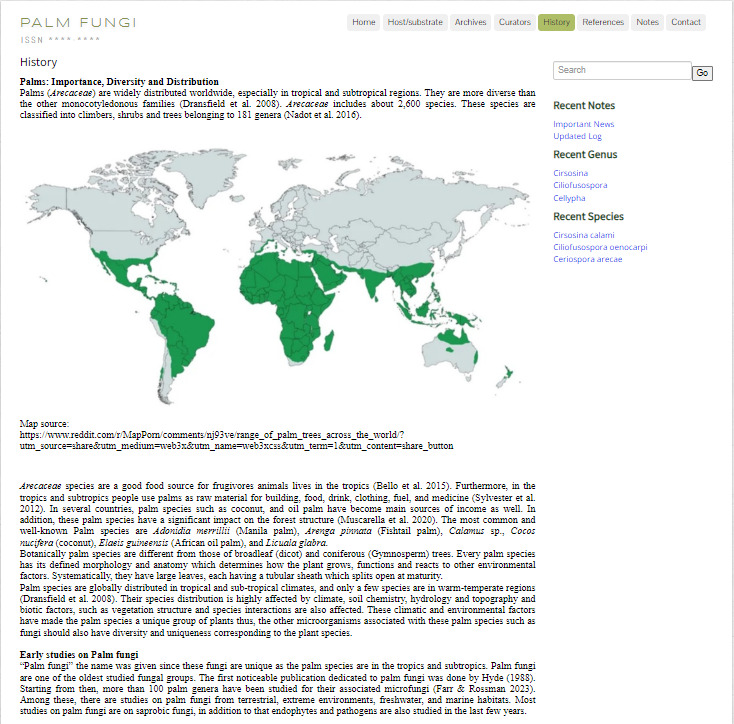
History: Website history information.

Shows the palm fungi collected, examined and recorded with a brief historical background (Fig. 9).

### References

**Figure 10.**
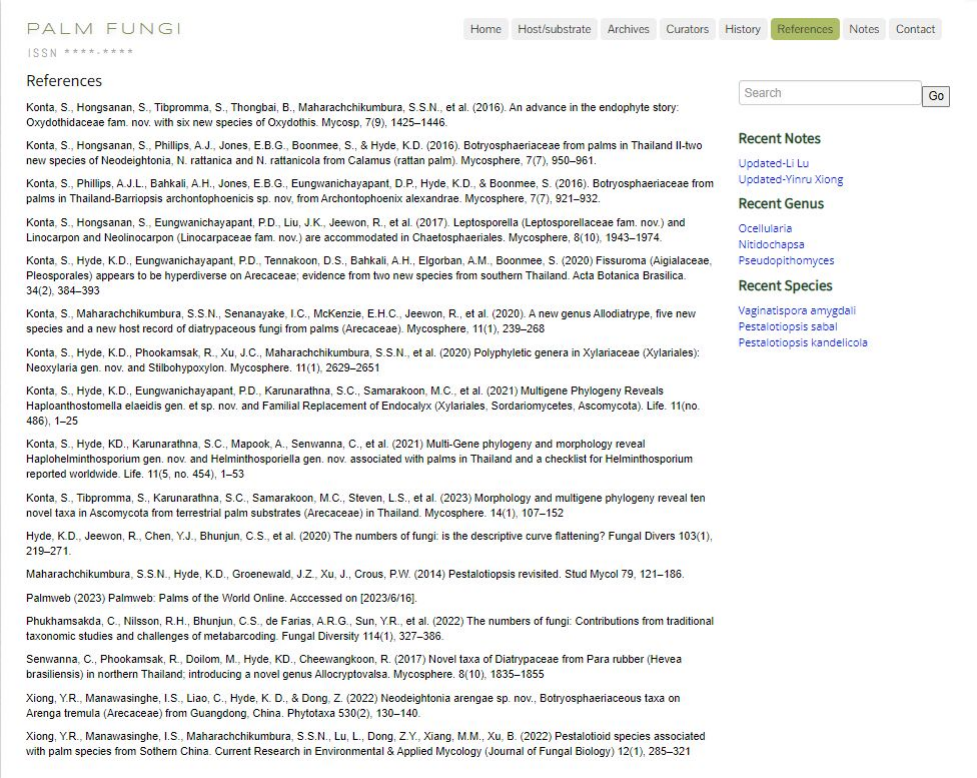
References: Website reference information.

Assembly of the main literature (such as books, reviews, monographs and articles) and websites (Fig. 10).

### Notes

**Figure 11.**
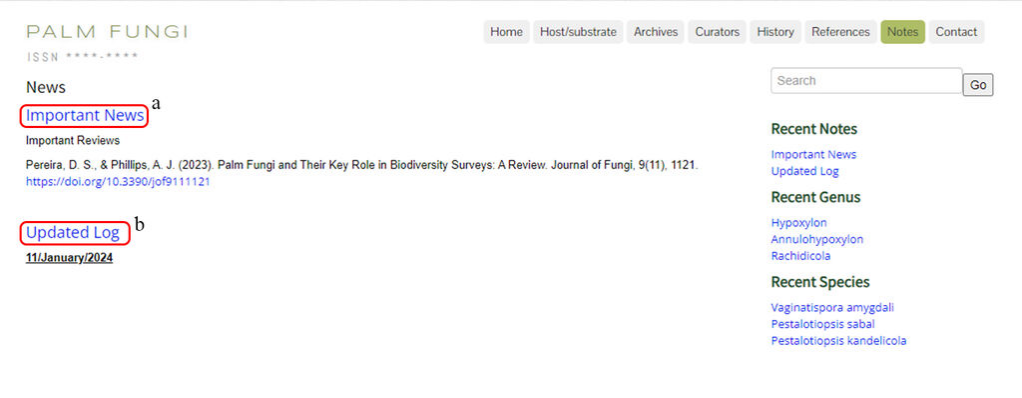
Notes: **a** Important News; **b** Updated Log.

The note section is dedicated to additional details of relevant to the palm fungi, with two sections (Fig. 11). One link is for Important News (a) and will link the recent reviews, publications and events or other updated news relevant to the palm fungi. The second is Updated Log (b) which shows the updated time and person who updated the website and includes the hyperlink of the update log.

### Contact

**Figure 12.**
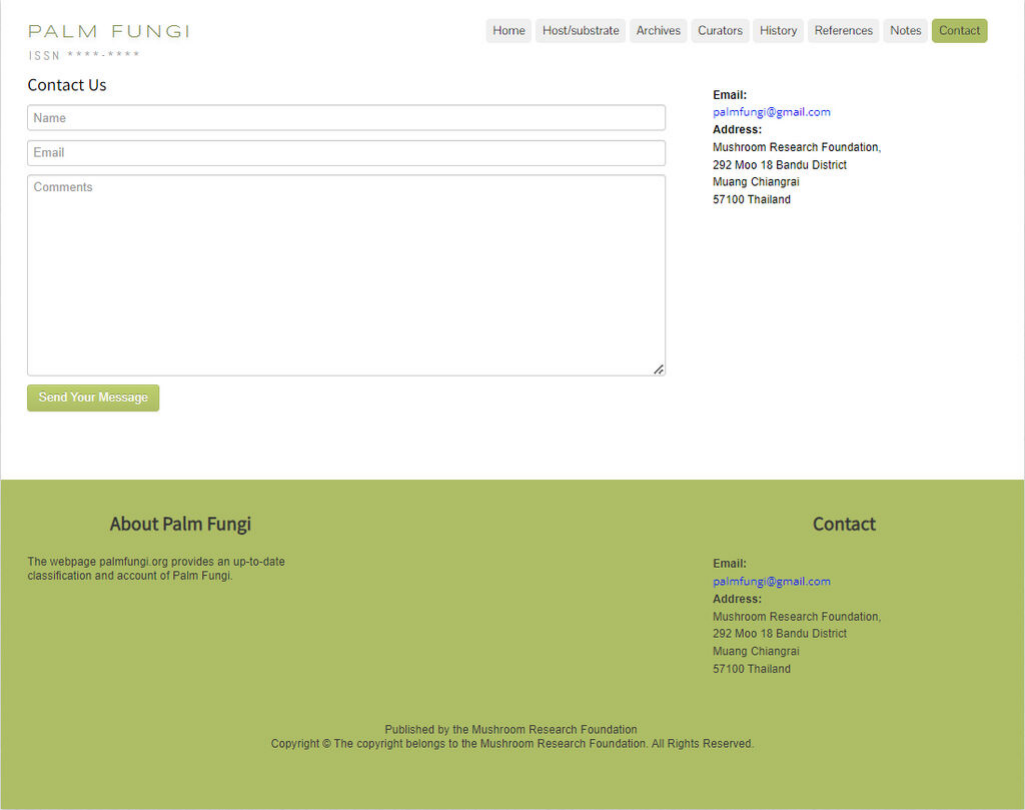
Contact: Website contact information.

Provides the contact information of the website and allows users provide any comments or suggestions (Fig. 12).

## Use palmfungi.org to briefly discuss the current status of research on the population distribution of palm fungi

Palm species have many uses (Dransfield et al. 2008), and Cámara-Leret et al. (2017) reported that 208 species of palm plants can be used as cash crops. Data from FAOSTAT (Food and Agriculture Organization of the United Nations 2024, https://www.fao.org/faostat/en/#data) also show that palms, especially oil palms and coconuts, have huge economic benefits. Based on data from Index Fungorum (2024) and Farr et al. (2021), palmfungi.org reviewed and included 1,521 fungal species associated with palm.

**Figure 13.**
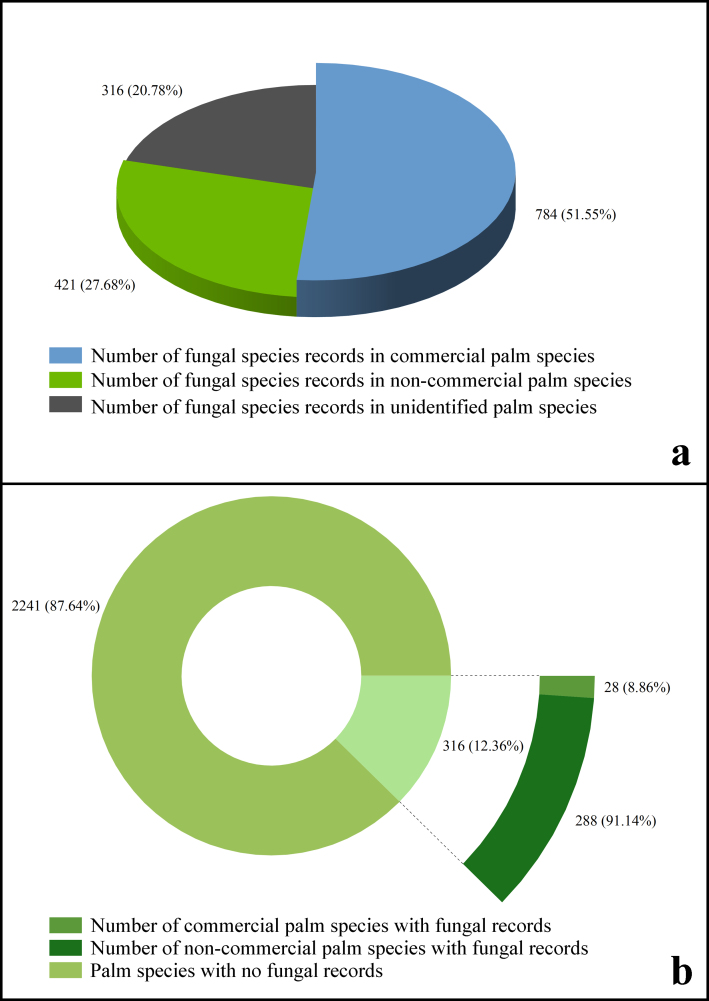
A diagram of numbers of palm fungi vs. number of palm species or Arecaceae.

Based on the above data, this study briefly analysed the reported distribution of fungi in the cash crops and non-cash crops of the palm. The results show in (Fig. 13a): 784 fungal species were reported from cash crop palm hosts, accounting for 51.55%; 421 fungal species were reported on non-cash crop palm hosts, accounting for 27.68%; 316 fungal species reported from unidentified palm species, accounting for 20.78%. Simultaneously, utilising the information from Host/Substrate in palmfungi.org, this study conducted a basic analysis to determine the number of palm species that have fungus records. The results showed as follows (Fig. 13b): there were 316 species of palm with fungi recorded, accounting for 12.36%, and 2241 species of Palm without fungi recorded, accounting for 87.64%.

Leveraging palmfungi.org as a platform, this study successfully conducted a basic analysis of the population distribution of palm fungi. The findings suggest that, while there is widespread documentation of palm fungi, the study focuses on a limited number of economically valuable palm species and overlooks the vast majority of naturally-occurring palm species.

## Discussion

The advancement of modern online platforms has greatly affected the ease of gathering information, which connects various databases about different biological information (Chaiwan et al. 2021). Basic information on related fungi can be found in several global fungal nomenclature and classification databases outlined in this paper. Those resources provides users with fungal host records and, as fungal species are continually being discovered, new host records and regional records are constantly being reported (Hyde et al. 2024). In recent years studies have recorded the fungal host-specificity (Zhou and Hyde 2001). Bubner (2013) reported the host-specificity of ectomycorrhizal fungi in pure and mixed stands of Scots pine (*Pinussylvestris* L.) and beech (*Fagussylvatica* L.), Miao et al. (2021) found that plant genetics are a deciding factor affecting *Nitrariatangutorum* endophytic fungal composition, Wang et al. (2019) pointed out that soil plant pathogenic fungi are specialised in low-level host taxa and Li et al. (2020) describe and discuss host-specific genes in most fungi at the molecular level. In addition, Hyde et al. (2007) pointed out that there are also differences in fungi from different locations on the same host. Despite the fact that fungi are so closely associated with plants, the only fungal-plant database available is for fungi on rice (https://mycolab.pp.nchu.edu.tw/rice_fungi/contact.php). However, this website cannot be used to explore fungal host specificity and is not interlinked with other large databases. The brief discussion of the distribution of palm fungi populations in this study fully demonstrates the effective application of palmfungi.org after interconnection with other large databases. Therefore, the operation of palmfungi.org provides a platform for the exploration of the host-fungus relationship.

We have presently uploaded 50 species entries to palmfungi.org and the website content will be continuously updated with the assistance of all curators. In the future, the palm fungi website will establish links between large and small fungal classification websites to form an integrated and interactive data collection platform. More attention will be paid to links with palm-related taxonomic websites such as palm web (https://palmweb.org), where associations with palm species will further improve the understanding of the relationship between fungi and palm hosts. Palmfungi.org will also be the first retrieval platform to record and retrieve taxonomic data on fungi and their specifically corresponding palm hosts. This platform will enable a new direction for exploring the host specificity of palm fungi and even the whole fungal kingdom.

## Figures and Tables

**Table 1.** List of expert curators with their contact information.

Position	Name	Address	Contact information
Head Curators	Kevin D. Hyde	Center of Excellence in Fungal Research, School of Science Mae Fah Luang University, Chiang Rai, Thailand 57100	kdhyde3@gmail.com
	Ishara S. Manawasinghe	Innovative Institute for Plant Health, Zhongkai University of Agriculture and Engineering, Guangzhou 510225, P.R. China	ishara9017@gmail.com
Managing curator	Yinru Xiong	Center of Excellence in Fungal Research, School of Science, Mae Fah Luang University, Chiang Rai, 57100, Thailand	richard_xyr@163.com
Curators	Joanne E. Taylor	Royal Botanic Garden Edinburgh, 20A Inverleith Row, Edinburgh EH3 5LR, United Kingdom	drjotaylor@yahoo.co.uk
	Alan J.L. Phillips	Biosystems and Integrative Sciences Institute, Faculdade de Ciências, 1749-016 Lisboa, Portugal	alan.jl.phillips@gmail.com
	Diana Santos Pereira	Biosystems and Integrative Sciences Institute, Faculdade de Ciências, 1749-016 Lisboa, Portugal	santospereira.dsp@gmail.com
	Shengnan Zhang	School of Life Science and Technology, Center for Informational Biology, University of Electronic Science and Technology of China, Chengdu, 611731, P.R. China	zshengnanbio@gmail.com
